# Millet cultivation mitigates soil erosion in sloping farmland by regulating near-soil surface traits

**DOI:** 10.3389/fpls.2026.1891497

**Published:** 2026-08-31

**Authors:** Chong Yao, Gaohan Xu, Siyuan Chen, Haozheng Wen, Mengen Jiu, Yonghua Liu, Zhijia Gu, Ming Zhu, Faqi Wu

**Affiliations:** 1 School of Geographic Sciences, Xinyang Normal University, Xinyang, Henan, China; 2 School of Teacher Education, Xinyang Normal University, Xinyang, Henan, China; 3 College of Soil and Water Conservation Science and Engineering, Northwest A&F University, Xianyang, Shaanxi, China

**Keywords:** millet cultivation, near soil-surface traits, rainfall simulation, sloping farmland, soil erosion

## Abstract

**Introduction:**

Crop growth induces seasonal variations in the near-soil surface traits, including vegetation features, root systems, and soil properties. However, the influencing mechanisms of variations in the near-soil surface traits on soil erosion remain unclear in sloping farmland.

**Methods:**

Rainfall simulation experiments with slope gradients of 5.24%, 8.75%, 17.63%, and 26.79% were carried out on bare soil control (CK) and millet cultivation plots at different growth stages. Near-soil surface traits such as soil bulk density (BD), aggregate mean weight diameter (MWD), soil cohesion (Coh), soil organic matter (SOM) content, soil crust thickness (CT), critical shear stress (*τ*_c_), rill erodibility (*K*_r_), root mass density (RMD), root surface area density (RSAD), root length density (RLD), plant height (PH), and leaf area index (LAI) were measured.

**Results:**

The results indicate that time-dependent dynamics of the near-soil surface traits exhibited seasonal variations with weak to strong variability. The initial runoff generation time (TR) was gradually advanced with the slope gradient and was delayed with millet growth. The mean runoff rate (RR), sediment rate (SR), and infiltration rate (IR) ranged 0.32–0.92 L m^−2^ min^−1^, 0.64–6.16 g m^−2^ min^−1^, and 0.43–1.09 mm m^−2^ min^−1^, respectively, during the entire growing season in the millet cultivation plots. The runoff reduction benefits (RRB) and sediment reduction benefits (SRB) ranged from 4.16% to 64.79% and from 17.35% to 93.18%, respectively. The interaction effects of soil properties, root system, and vegetation features accounted for the variations in the total runoff volume of 76.76%, sediment yield of 82.61%, and total infiltration of 85.71%. The sediment yield decreased with the improvement of the soil properties and the development of the root system as exponential or linear relationships.

**Conclusion:**

The results demonstrate seasonal variations in the near-soil surface traits induced by crop growth and provide reliable support for soil erosion control and sustainable development in cropland.

## 1 Introduction

Soil erosion is one of the major environmental problems globally, causing financial losses and sediment accumulation in rivers (Futerman et al., 2025; Quinton et al., 2010; Saadati et al., 2023). Due to the complex geological and topographical conditions and long-term intensive agricultural cultivation, the Loess Plateau is one of the regions with the most severe soil erosion (Song et al., 2026; Tuo et al., 2025; Yao et al., 2025; Zhao et al., 2023). The sloping farmland makes a significant contribution to sediment generation in the Loess Plateau, accounting for approximately 60% of the total (Wang et al., 2017). Soil erosion is generally regarded as being at a high-intensity level all year on sloping farmland in the Loess Plateau (Lin et al., 2019; Wang et al., 2017). However, recent studies have shown that the soil detachment capacity in sloping farmland exhibits remarkable seasonal variations (Yao et al., 2022). Thus far, the seasonal dynamics of soil erosion and its underlying mechanisms remain underexplored in sloping farmland. This is particularly crucial for quantifying the intricate interplay between crop-induced variations in soil surface traits and soil erosion.

The aboveground components of plants mitigate soil erosion through a shielding effect against rainfall and by slowing down the runoff velocity (Ludwig et al., 2006; Puigdefábregas, 2005; Wang et al., 2024). Crop canopy reduces raindrop splash more than 50% by mitigating the throughfall intensity and lowering the kinetic energy of rainfall (Ma et al., 2015). Crop residues and soil are mixed to form a straw–soil matrix, altering the soil permeability and porosity (Chen et al., 2025; Zhang et al., 2025b). At the same time, the effects of the straw–soil matrix and straw mulching on soil loss also vary with coverage and crop growth (Wang et al., 2022, 2023). Plant mitigates the precipitation concentration by allowing it to pass along the stems and reach the soil surface, minimizing the direct raindrop impact on soil mass (Ma et al., 2016; Van Dijk and Bruijnzeel, 2001). Due to the existence of plant stems, the soil surface roughness is increased and the flow velocity is reduced (Bautista et al., 2007; Di Prima et al., 2026). Crop canopy varies with crop growth, and the retardation effects of the plant’s aboveground components on soil erosion during the different growth stages need to be further investigated.

The plant root systems mitigate soil erosion through chemical bonding and physical binding (Gyssels et al., 2005; Knapen et al., 2007b; Wang and Zhang, 2017). The plant root systems spread within the soil in all directions, forming root–soil complexes through interweaving, entangling, and adhering to the soil mass (Chen et al., 2024; Vannoppen et al., 2015). The formation of root–soil complexes and network structures enhance soil erosion resistance by more than 20% and vary with root type and root biomass (Geng et al., 2015; Jimenez Aguilar et al., 2009; Li et al., 2015; Nandintsetseg and Shinoda, 2011; Yao et al., 2022). The soil erosion reduction benefits provided by root systems are closely related to the root mass density (RMD), root surface area density (RSAD), and root length density (RLD) (Burylo et al., 2012; Chen et al., 2024; Loades et al., 2010; Zhou et al., 2025). In general, soil erosion decreases with root traits following an exponential or a power function (Yao et al., 2023). Although the quantitative relationship between soil erosion and root traits has been investigated, there is still a need to investigate variations in quantitative relationship with crop growth.

Changes in the soil properties influence the soil erosion process by regulating soil stability and soil erosion resistance. At the same time, crop root systems also regulate soil properties through root development and by providing nutrients to the soil (Ma et al., 2021; Nardi et al., 2000). The binding material and organic matter released by the roots facilitate the formation of water-stable aggregates and the accumulation of soil organic matter (SOM) content (Li et al., 2017; Yao et al., 2022; Zhou et al., 2025). The roots squeeze the soil, creating micro-channels in the soil mass, thus enhancing the soil permeability and influencing the soil bulk density (BD) (Bahddou et al., 2023; Zhang et al., 2025a). Due to the formation of the soil–root complex, the soil cohesion (Coh) and soil shear strength are improved (Zi et al., 2023). In contrast to the bare soil control (CK), the soil erosion resistance (critical shear stress and rill erodibility) decreases by 16.00% to 61.36% during one growing season. The formation of soil crust by rainfall impact and the blockage and sealing of the soil pores also enhance soil shear strength. Numerous studies have found that the soil anti-erosion ability shows significant seasonal variations triggered by crop growth (Yao et al., 2022). The mutual effects between soil properties and root systems vary seasonally with crop growth; however, it still remains unclear how these effects regulate soil erosion.

Severe soil erosion occurs on the Loess Plateau, with an annual erosion rate of 5,000–10,000 ton km^−2^ (Ma et al., 2022; Wang et al., 2025c; Zhang et al., 2017). Due to multilayered protective systems and less human disturbance, forests and natural grasslands exhibit stronger soil erosion prevention functions (Chen et al., 2024; Di Prima et al., 2026). As a result of frequent human disturbances and the single vegetation structure, soil erosion is at a relatively high level in sloping farmlands (Jiang et al., 2025; Ma et al., 2021). Due to the dry climate in the Loess Plateau, millet, as a traditional dryland crop, possesses excellent drought resistance and tolerance to poor soil conditions (Yao et al., 2022). Therefore, millet is one of the main crop types in the sloping farmland on the Loess Plateau. Thus far, only a few research works have quantified the impact of millet growth on the near-soil surface traits represented by vegetation features, root systems, and soil properties. Furthermore, the relationship between soil erosion and near-soil surface traits in sloping farmlands is not fully understood.

Thus, rainfall simulation was carried out in millet cultivation in runoff plots and CK plots and the near-soil surface traits measured across different growing seasons. The aims of our work were to: 1) quantify the variations in the near-soil surface traits triggered by millet cultivation during the entire growing season; 2) investigate changes in the soil erosion process and the runoff reduction benefits (RRB) and sediment reduction benefits (SRB) with millet growth; and 3) determine the relationship between soil erosion and near-soil surface traits and identify the main influencing factors.

## 2 Materials and methods

### 2.1 Study area

The current study was conducted in Yangling, Shaanxi Province (34°18′ N, 108°07′ E), located in the southern part of the Loess Plateau. This area has a warm temperate semi-humid continental climate. The annual precipitation is 600–800 mm, which is mainly concentrated in the rainy season (from July to September). The average annual temperature is 12.9°C, with a frost-free period of 211 days and with 2,163.8 h of average annual sunshine. This region has abundant sunshine and moderate rainfall, with huge potential for agricultural development. It is the birthplace of Chinese civilization and one of the traditional agricultural areas in China. The cultivated land in this area is mainly a gentle slope (<26.79%), and the main food crops include wheat, corn, millet, and soybean. Millet is generally sown in strip planting at the end of May, with a growth period of approximately 100–115 days. The test soil had sand, silt, and clay contents of 30.20%, 41.60%, and 28.20%, respectively.

### 2.2 Rainfall simulator

**Figure 1:**
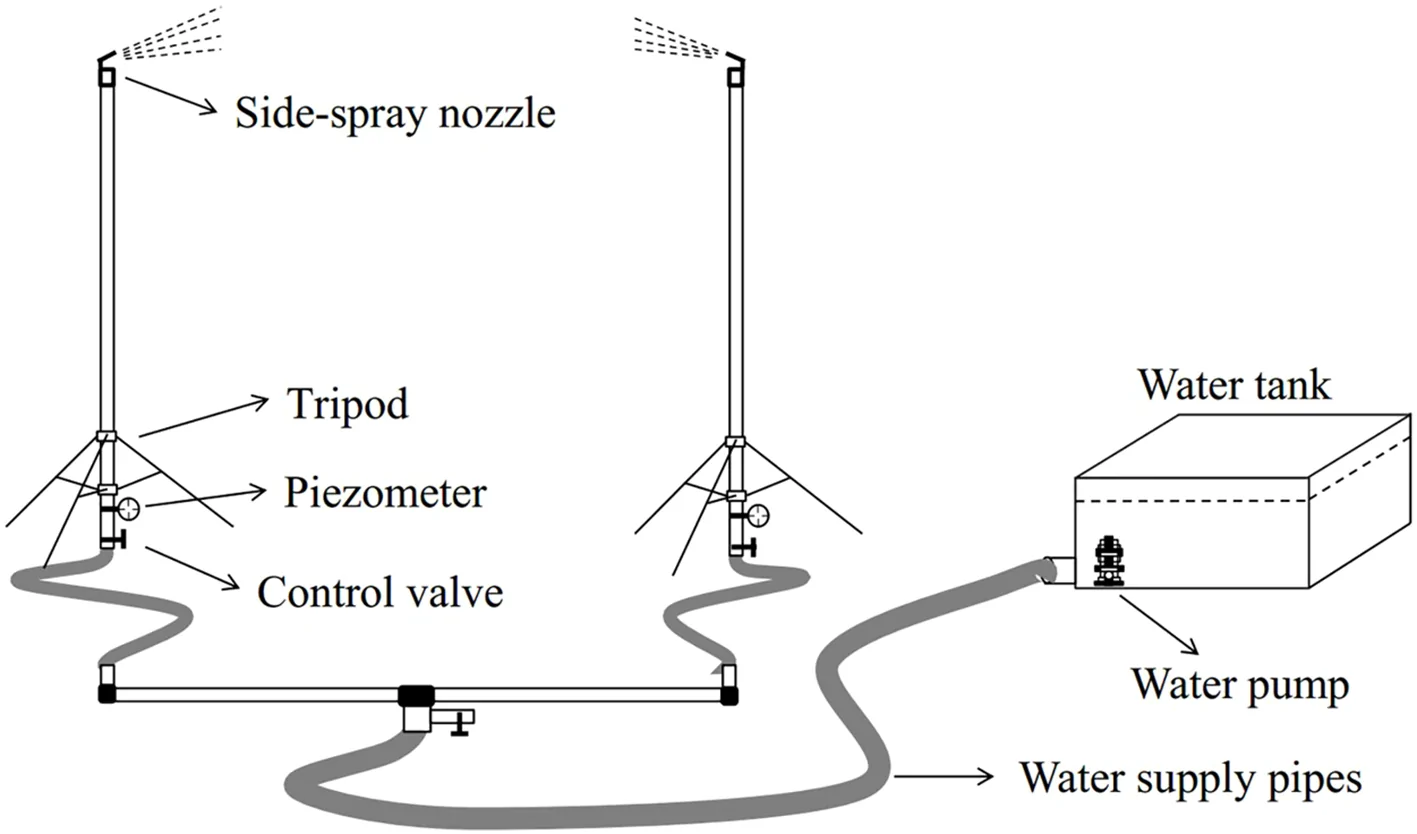
Schematic of the side-spray rainfall simulation system.

The rainfall simulator involves a water supply system, pressure control valve, tripod, rainfall support frame, rainfall nozzles water tank, water pump, and water supply pipeline (**Figure 1**). The rainfall simulator has a rainfall height of 8 m, with an effective rainfall area of 7 m × 5 m and rainfall uniformity of more than 85%. The simulated raindrop size distribution of 0.2–3.6 mm is basically consistent with a natural raindrop distribution. The water used for the experimental rainfall is tap water. The water supply equipment and the rainfall simulator could meet a rainfall intensity range of 30–150 mm h^−1^.

### 2.3 Runoff plot and experiment design

A runoff plot with length of 5.0 m, width of 1.4 m, and depth of 0.6 m was used for millet cultivation and for CK. The test soil was collected from the 0- to 20-cm layer of local sloping farmland and was air-dried and sieved through a 10-mm screen. The bottom of the runoff plots was filled with a 5-cm layer of fine sand to facilitate free drainage. The runoff plots were filled in layers to ensure that the soil BD was consistent with that of the sloping farmland (1.15–1.20 g cm^−3^). Each layer was gently leveled with a wooden board after filling to eliminate discontinuities between the soil layers. After soil filling was completed, the plots were left for 15 days.

The millet seeds chosen were Jin Gu 21 seeds. Rainfall simulation was carried out at the seeding (S1), jointing (S2), flowering (S3), filling (S4), and ripening (S5) stages and the CK. Based on long-term natural rainfall observations, topographical conditions, and actual situation in the sloping farmland, a rainfall intensity of 80 mm h^−1^ and slope gradients of 5.24%, 8.75%, 17.63%, and 26.79% were designed for the experiment.

### 2.4 Observation of the soil erosion process and measurement of the near-soil surface traits

After rainfall simulation commenced, the initial runoff generation time (*T*_R_) was recorded. The sediment sample was collected every 3 min and the weight (*S*_a_, in grams) was recorded. The collected samples were dried and the final dry mass (*S*_b_, in grams) was recorded. According to the works of Lin et al. (2019) and Wang et al. (2017), the sediment rate (SR, in grams per square meter per minute), the runoff rate (RR, in liters per square meter per minute), and the infiltration rate (IR, in liters per square meter per minute) were calculated using Equations 1, 2, and 3, respectively.

(1) $$SR=\frac{Sb}{At}$$

(2) $$IR=\frac{Sa-Sb}{1000At}$$

(3) $$RR=\frac{I-h}{At}$$

where *A*, *t*, *I*, and *h* are the area of the runoff plot (in square meters), the sampling time (in minutes), the rainfall intensity (in millimeters per minute), and the runoff depth (in millimeters), respectively.

Soil samples were collected using ring knives with a volume of 100 cm^3^ to determine the soil BD with 10 repetitions during the different growing seasons. The mean weight diameter (MWD) was measured using the improved wet sieving method with 10 repetitions (Yoder, 1936). An Eijkelkamp pocket vane tester (Eijkelkamp, Giesbeek, the Netherlands) was used for Coh with 20 replicates. The SOM content was determined using the potassium dichromate colorimetric method with 10 repetitions. The soil crust thickness (CT) was measured using Vernier calipers with a measurement accuracy of 0.01 mm and with 20 replicates. Soil samples were collected using ring knives with a volume of 500 cm^3^ during the different growing seasons and were rinsed with low water pressure to separate the roots from the soil matrix. Subsequently, the separated root was scanned using a root scanner. According to the work by Wang et al. (2025c), the root was dried and the dry mass recorded as *M*_r_ (in grams). The RLD (in centimeters per cubic centimeter), RSAD (in square centimeters per cubic centimeter), and RMD (in kilograms per cubic meter) were calculated using Equations 4, 5, and 6, respectively.

(4) $$RMD=\frac{Mr}{Vs}$$

(5) $$RLD=\frac{Lr}{Vs}$$

(6) $$RSAD=\frac{Rsa}{Vs}$$

where *V*_s_, *L*_r_, and *R*_sa_ are the volume of the ring knife (in cubic centimeters), the root length (in centimeters), and the root surface area (in square centimeters), respectively. The critical shear stress (*τ*_c_, in pascals) and rill erodibility (*K*_r_, in seconds per meter) were calculated using the Water Erosion Prediction Project (WEPP) model. The height of the millet (PH) was measured using a rule, recording the distance from the top of the plants to the ground. The leaf area index (LAI) was measured with the image method.

### 2.5 Statistical analyses

Differences in the near-soil surface traits among the different growth stages in the millet cultivation and CK plots were assessed using ANOVA. The correlation between the near-soil surface characteristics (e.g., vegetation features, root systems, and soil properties) and the soil erosion indicators was examined using Pearson’s correlation analysis. The quantitative relationships between the near-soil surface traits and the soil erosion indicators were quantified using a regression analysis model. Variance partitioning analysis was employed to quantify the impact of near-soil surface traits on soil erosion.

## 3 Results

### 3.1 Near-soil surface traits

**Figure 2:**
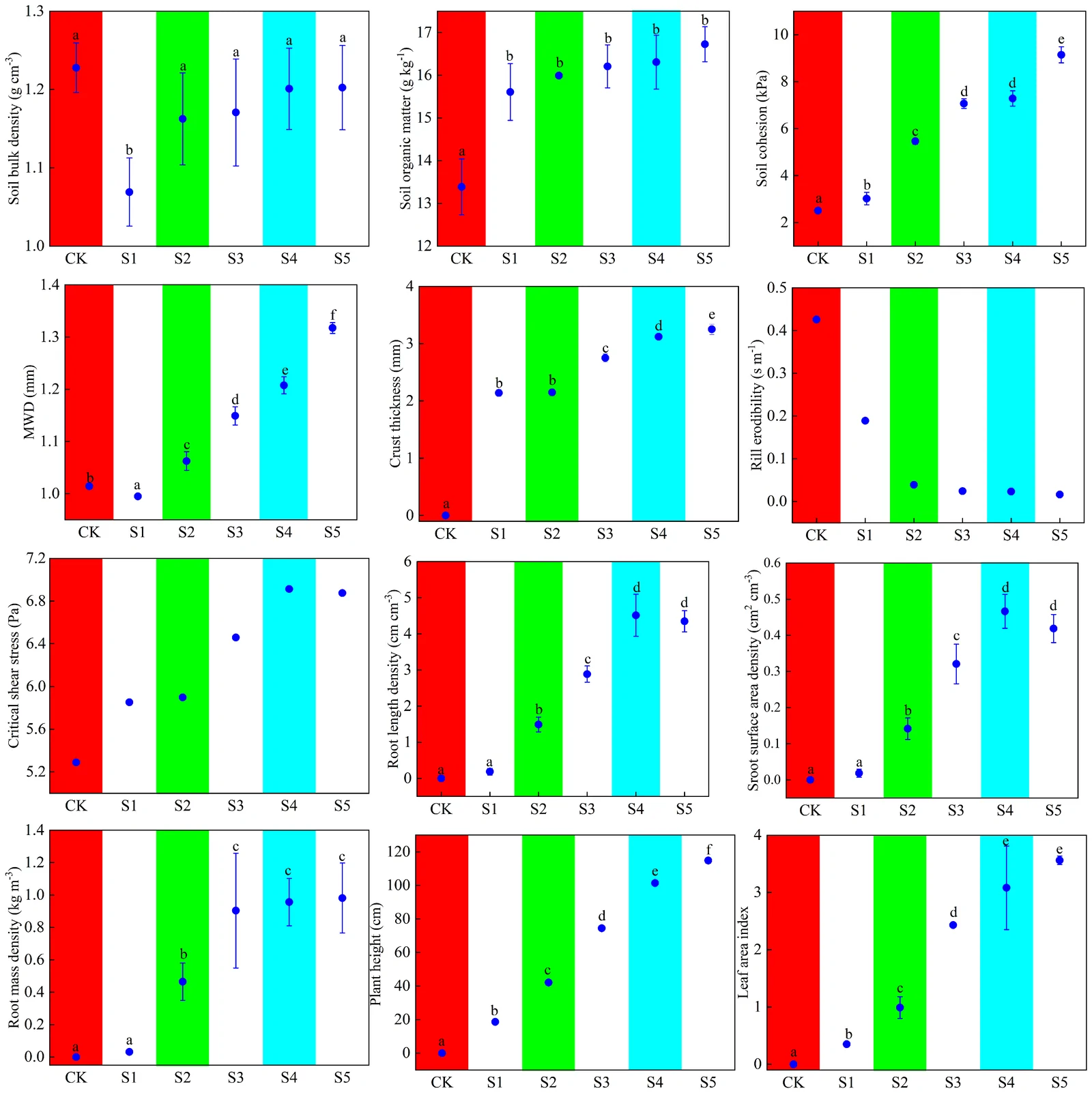
Variations in the near-soil surface traits in a millet growing season. *Different lowercase letters* indicate significant differences in the near-soil surface traits among the different growth stages. *CK*, bare soil control; *S1*, seeding stage; *S2*, jointing stage; *S3*, flowering stage; *S4*, filling stage; *S5*, ripening stage.

The near-soil surface traits and their seasonal variations in the millet cultivation and CK plots are presented in **Figure 2**. The LAI and PH increased over time and ranged 0.35–3.56 and 18.65–114.83 cm, respectively, from S1 to S5. The BD for the millet cultivation plots was lower than that for the CK plot, but gradually increased with extension of the growing season. The Coh and MWD for the millet cultivation plots were higher than those for the CK plots and gradually increased with millet growth. SOM increased from 15.61 to 16.72 g kg^−1^. The RMD increased steadily with crop growth, from 0.03 kg m^−3^ in S1 to 0.98 kg m^−3^ in S5. The RLD and RSAD increased first and then decreased with millet growth, reaching the maximum at S4, which were 4.51 cm cm^−3^ and 0.47 cm^2^ cm^−3^, respectively. Rill erodibility in the millet cultivation plot was lower than that in CK and decreased with crop growth. In contrast, the *τ*_c_ increased with crop growth, ranging from 5.85 Pa in S1 to 6.87 Pa in S5. Compared with that in S1, the BD, Coh, SOM, MWD, CT, and *τ*_c_ increased by 8.75%–12.48%, 80.72%–202.41%, 2.45%–7.17%, 6.80–32.43%, 0.47%–51.87%, and 0.78–22.48%, respectively, and *K*_r_ decreased by 79.37%–91.53% from S2 to S5. In contrast to that in S1, the RLD, RSAD, and RMD increased by 7.05–22.18, 6.71–21.81, and 13.94–30.55 times, respectively, from S2 to S5. The variation coefficients of BD, SOM, and *τ*_c_ ranged from 0.03 to 0.08, demonstrating weak variability, while the variation coefficients of MWD, Coh, RLD, RSAD, RMD, PH, and LAI ranged from 0.11 to 0.69, indicating moderate variability. The variation coefficient of *K*_r_ was 1.26, showing strong variability.

### 3.2 Soil erosion process

#### 3.2.1 Initial runoff generation time

**Figure 3:**
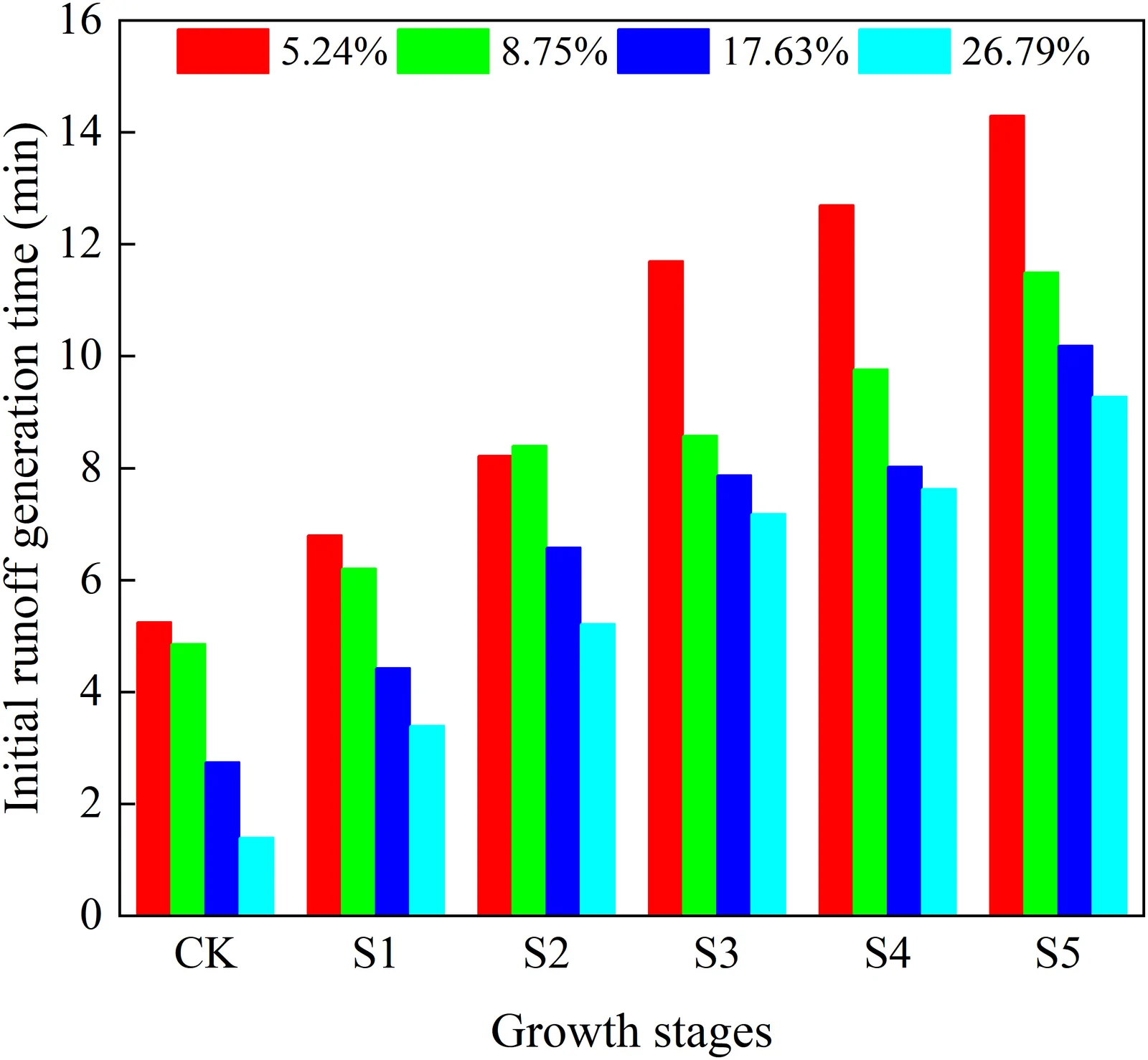
Variations in the initial runoff generation time (in minutes). *CK*, bare soil control; *S1*, seeding stage; *S2*, jointing stage; *S3*, flowering stage; *S4*, filling stage; *S5*, ripening stage.

The initial *T*_R_ in the millet cultivation and CK plots showed obvious changing trends with different slope gradients and crop growing seasons (**Figure 3**). The earliest initial *T*_R_ was produced in the CK plot on a slope gradient of 26.79%, with an initial *T*_R_ of 1.40 min. Under a slope gradient of 5.24%, the initial *T*_R_ in the millet cultivation plots extended from 6.80 min in S1 to 14.30 min in S5, while that for CK was 5.25 min. The initial *T*_R_ in the millet cultivation plots with a slope gradient of 26.79% extended from 3.40 min in S1 to 9.28 min in S5. Overall, the initial *T*_R_ in the CK and millet cultivation plots was delayed with millet growth and advanced with slope gradient.

#### 3.2.2 Runoff rate

**Figure 4:**
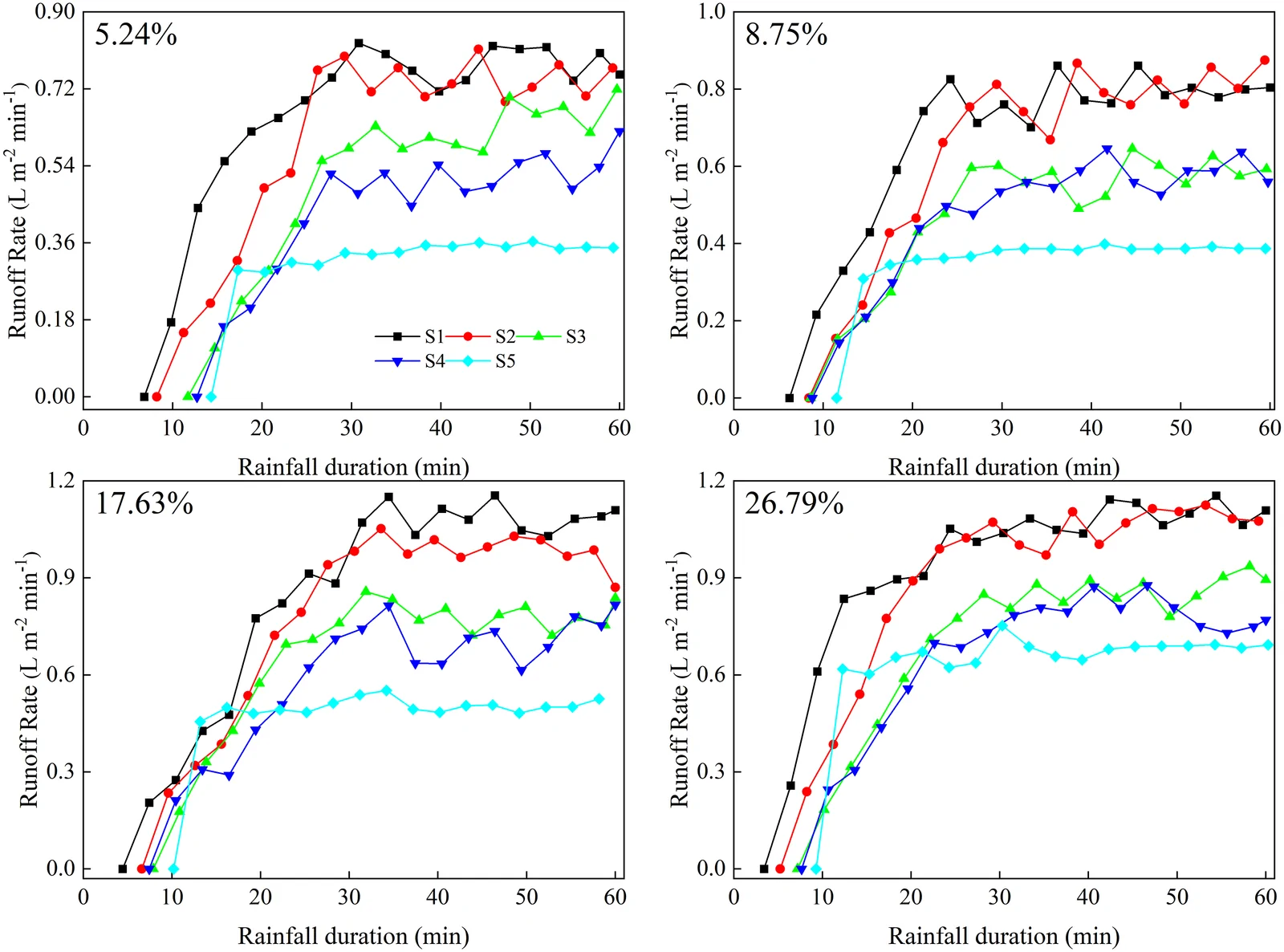
Variations in the runoff rate with rainfall duration for the different millet growth stages. *S1*, seeding stage; *S2*, jointing stage; *S3*, flowering stage; *S4*, filling stage; *S5*, ripening stage.

As shown in the **Figure 4**, the RR decreased with crop growth in the millet cultivation plots. The mean RR at a slope gradient of 5.24% in S1 to S5 ranged from 0.32 to 0.66 L m^−2^ min^−1^. At a slope gradient of 5.24%, the mean RR in S5 decreased by 51.98%, which is in contrast to that in S1. The average RR with a slope gradient of 26.79% in S1 to S5 ranged from 0.63 to 0.92 L m^−2^ min^−1^. At a slope gradient of 26.79%, the average RR in S5 decreased by 31.36%, in contrast to that in S1. The average RR showed a downward trend with millet growth, indicating that the growth and development of millet could effectively delay the generation of surface runoff and reduce the RR. In S1, the mean RRs with slope gradients of 5.24%, 8.76%, 17.63%, and 26.79% were 0.66, 0.66, 0.84, and 0.92 L m^−2^ min^−1^, respectively. In S1, the average RR with a slope gradient of 26.79% increased by 40.16%, in contrast to a slope gradient of 5.24%. In S5, the mean RRs with slope gradients of 5.24%, 8.76%, 17.63%, and 26.79% were 0.32, 0.35, 0.47, and 0.63 L m^−2^ min^−1^, respectively. In S5, the average RR with a slope gradient of 26.79% increased by 100.36% compared with that with a slope gradient of 5.24%.

#### 3.2.3 Sediment rate

**Figure 5:**
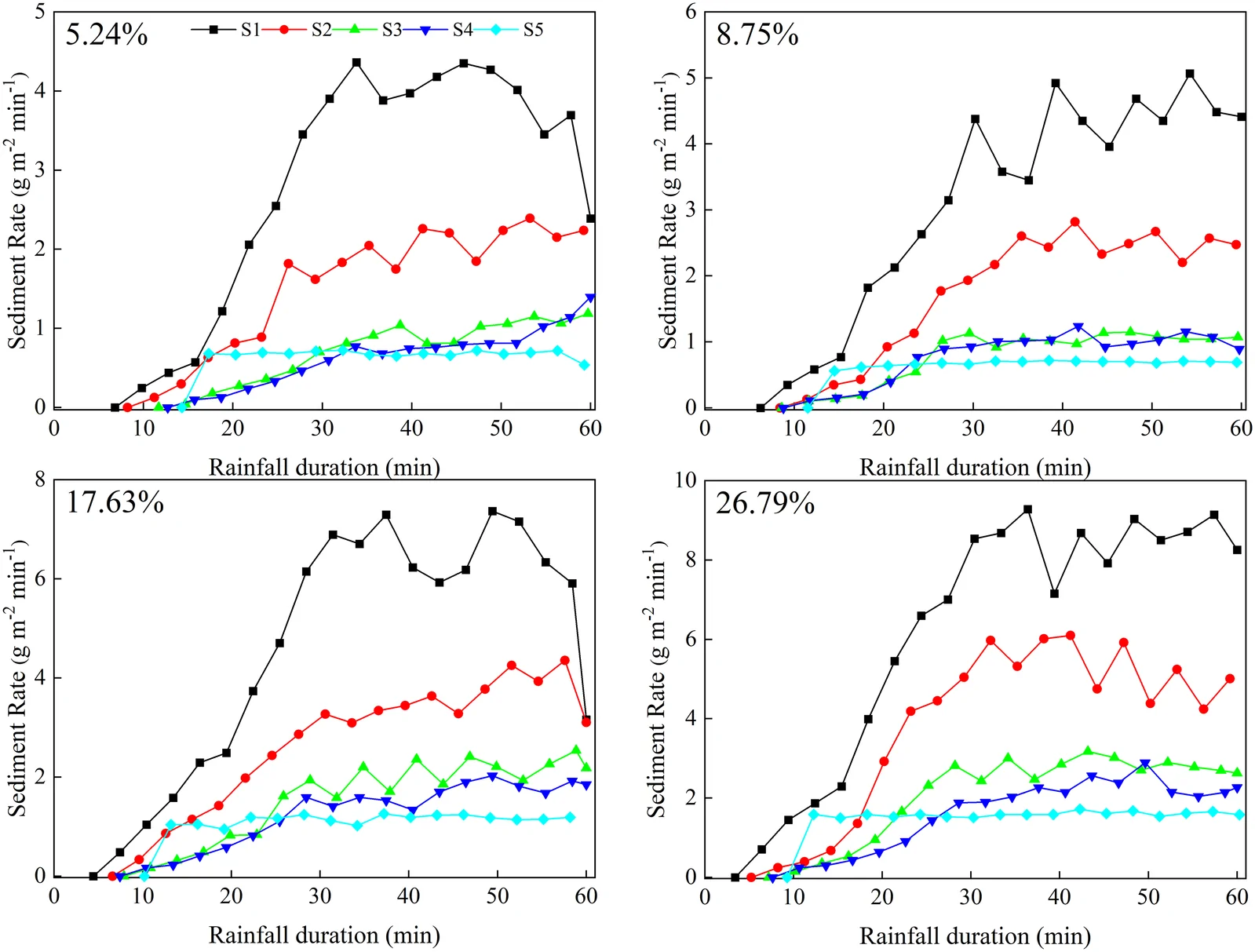
Variations in the sediment rate with rainfall duration for the different millet growth stages. *S1*, seeding stage; *S2*, jointing stage; *S3*, flowering stage; *S4*, filling stage; *S5*, ripening stage.

The SR gradually decreased with crop growth in the millet cultivation plots (**Figure 5**). The mean SRs at a slope gradient of 5.24% in S1, S2, S3, S4, and S5 were 2.79, 1.51, 0.70, 0.64, and 0.64 g m^−2^ min^−1^, respectively. At a slope gradient of 5.24%, the mean SR in S5 decreased by 77.21% compared with that in S1. In contrast to S1, the mean SR in S5 with slope gradients of 8.75%, 17.60%, and 26.79% decreased by 79.49%, 76.43%, and 75.66%, respectively. In S1, the mean SRs with slope gradients of 5.24%, 8.75%, 17.60%, and 26.79% were 2.79, 3.11, 4.58, and 6.16 g m^−2^ min^−1^, respectively. In S1, the average SR with a slope gradient of 26.79% increased by 120.95%, in contrast to that with a slope gradient of 5.24%. In S5, the mean SRs with slope gradients of 5.24%, 8.76%, 17.63%, and 26.79% were 0.64, 0.64, 1.08, and 1.50 g m^−2^ min^−1^, respectively. In contrast to that with a slope gradient of 5.24%, the mean SR with a slope gradient of 26.79% in S5 increased by 135.96%. The average SR with a slope gradient of 26.79% in growth stages S2, S3, and S4 increased by 152.20%, 196.89%, and 153.70%, respectively, compared with 5.24%. This indicates that the slope gradient had a significant promoting effect on the SR in sloping farmland.

#### 3.2.4 Infiltration rate

**Figure 6:**
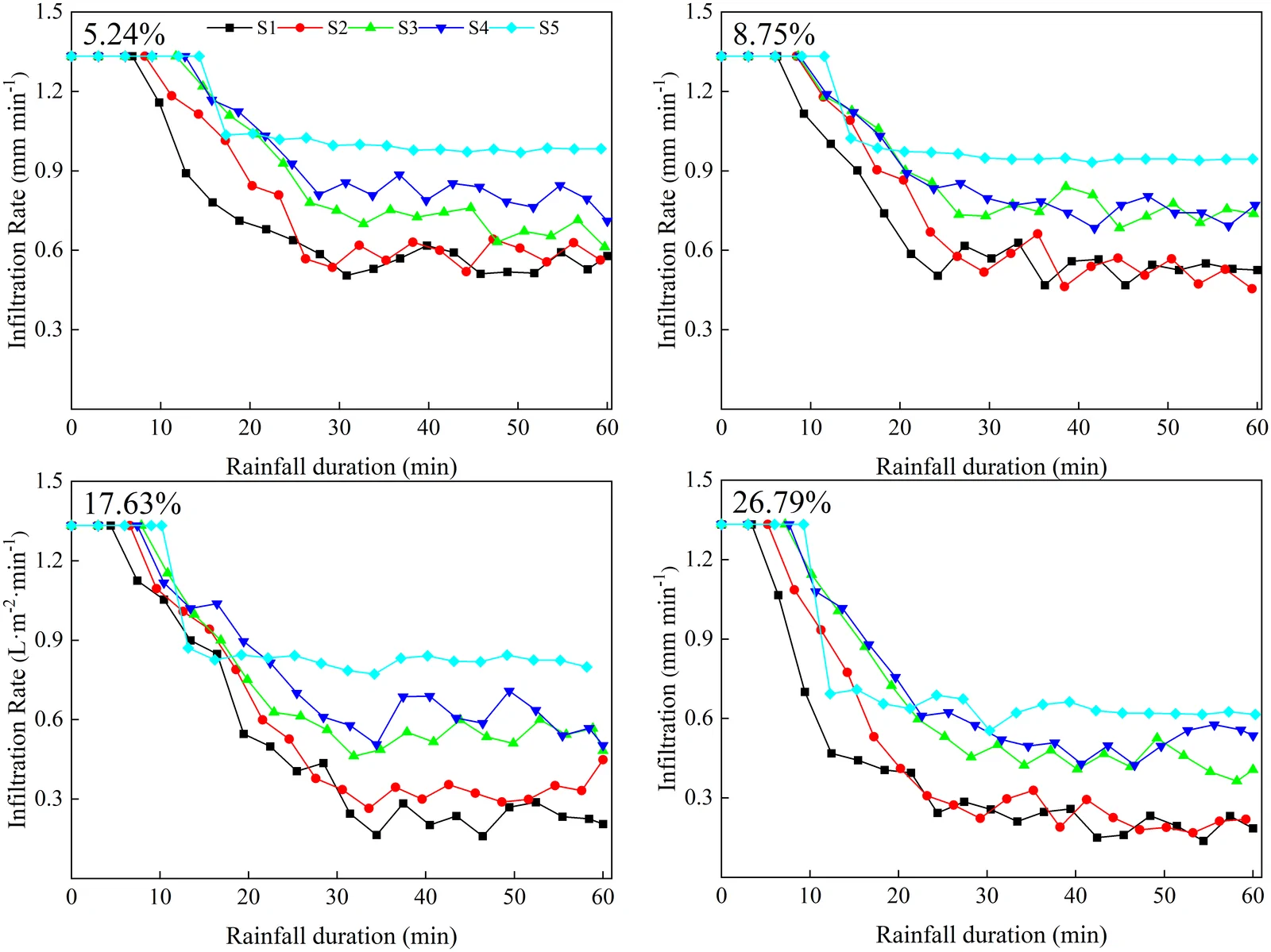
Variations in the infiltration rate with rainfall duration for the different millet growth stages. *S1*, seeding stage; *S2*, jointing stage; *S3*, flowering stage; *S4*, filling stage; *S5*, ripening stage.

The IR in the millet cultivation plots maintained a relatively high level at the beginning of rainfall, which then fluctuated and decreased with rainfall duration. As shown in **Figure 6**, taking a slope gradient of 5.24% as an example, the average IRs ranged from 0.74 to 1.09 mm^−2^ min^−1^ in S1 to S5, respectively. The mean IR in S5 increased by 47.97% relative to that in S1 at a slope gradient of 5.24%. The average IRs in S1 to S5 with a slope gradient of 26.79% ranged from 0.43 to 0.77 mm^−2^ min^−1^, respectively. In contrast to that in S1, the average IR in S5 increased by 81.40% under a slope gradient of 26.79%. In S1, the average IRs with slope gradients of 5.24% and 26.79% were 0.43 and 0.74 mm min^−1^, respectively. In S1, the mean IR with a slope gradient of 26.79% was 42.31% lower than that with a slope gradient of 5.24%. During growth periods S2, S3, S4, and S5, the average IRs with a slope gradient of 26.79% were 37.43%, 27.13%, 26.72%, and 29.28% lower than those at a slope gradient of 5.24%, respectively. The IR in the millet cultivation plots gradually increased with crop growth and decreased with slope gradient.

### 3.3 Runoff and sediment reduction benefits

**Figure 7:**
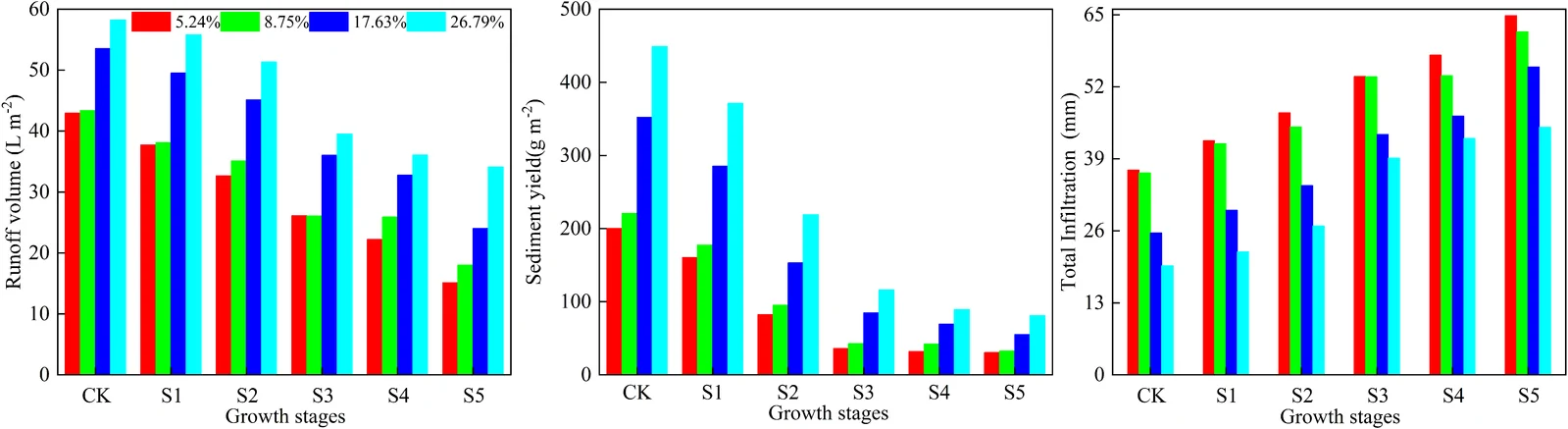
Variations in the total runoff volume, total sediment yield, and total infiltration for the different soybean growth stages. *CK*, bare soil control; *S1*, seeding stage; *S2*, jointing stage; *S3*, flowering stage; *S4*, filling stage; *S5*, ripening stage.

In the current study, the total runoff volumes with slope gradients of 5.24%, 8.75%, 17.60%, and 26.79% in the CK plots were 42.97, 43.38, 53.55, and 58.25 L m^−2^, respectively, and the sediment yields were 200.33, 220.67, 352.33, and 449.00 g m^−2^, respectively. The RRB and SRB in the millet cultivation plots at different slopes and growing seasons were calculated (**Table 1**). The total runoff volumes, sediment yields, and total infiltration are plotted in **Figure 7**. The results indicated that the RRB values with a slope gradient of 5.24% in S1 and S5 were 12.27% and 64.79%, respectively. The RRB values with slope gradients of 8.75%, 17.60%, and 26.79% in different growth stages ranged 12.14%–58.47%, 7.50%–55.10%, and 4.16%–41.43%, respectively. Overall, the RRB decreased with slope gradient and increased with the extension of the growing season. The SRB with slope gradients of 5.24%, 8.75%, 17.60%, and 26.79% ranged 19.90%–92.45%, 19.55%–91.84%, 19.00%–93.18%, and 17.35%–92.40%, respectively. The SRB increased with the extension of the growing season. Overall, the SRB values were generally higher than the RRB.

**Table 1:** Runoff reduction benefits (RRB), sediment reduction benefits (SRB) for different crop growing seasons and slope gradients.

Growth stage	RRB				SRB			
	5.24%	8.75%	17.63%	26.79%	5.24%	8.75%	17.63%	26.79%
S1	12.27	12.14	7.50	4.16	19.90	19.55	19.00	17.35
S2	23.97	19.07	15.67	11.88	58.94	56.74	56.51	51.21
S3	39.24	39.87	32.69	32.16	81.93	80.62	75.90	74.16
S4	48.24	40.29	38.81	38.06	84.00	80.92	80.29	80.14
S5	64.79	58.47	55.10	41.43	92.45	91.84	93.18	92.40

*S1*, seeding stage; *S2*, jointing stage; *S3*, flowering stage; *S4*, filling stage; *S5*, ripening stage.

### 3.4 Correlation between near-soil surface traits and soil erosion indicators

**Figure 8:**
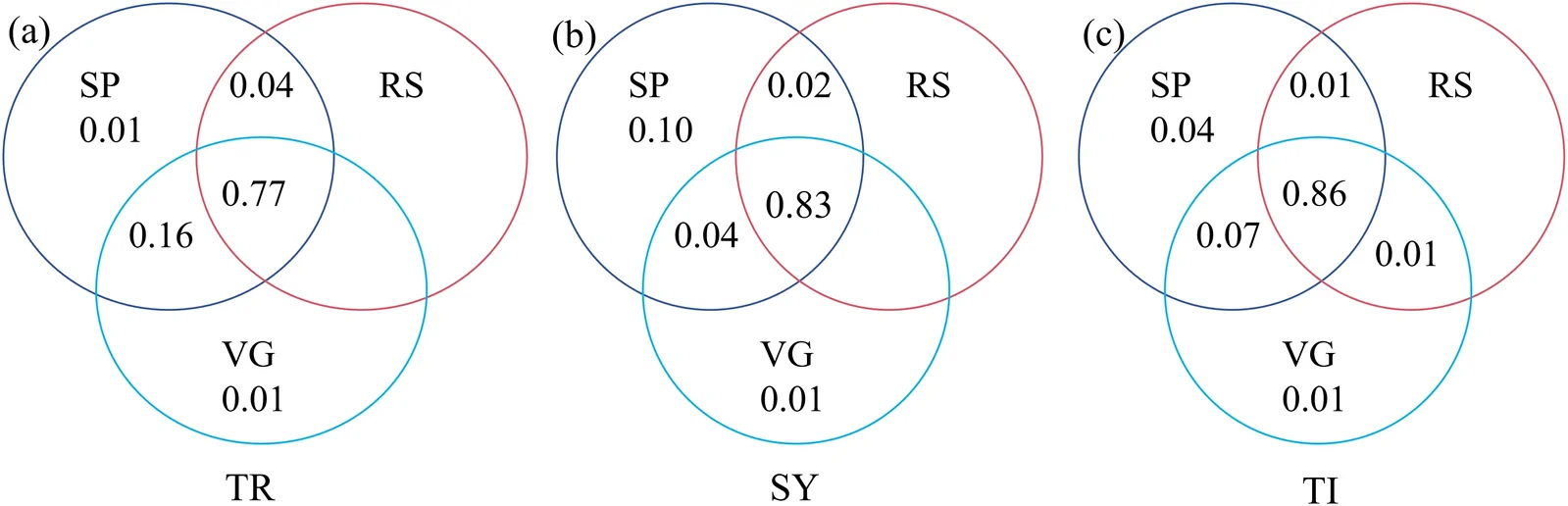
Relative effects of soil properties (*SP*), root traits (*RS*), and vegetation features (*VG*) on the total runoff volume (*TR*) **(a)**, total sediment yield (*SY*) **(b)**, and total infiltration (*TI*) **(c)**.

**Figure 9:**
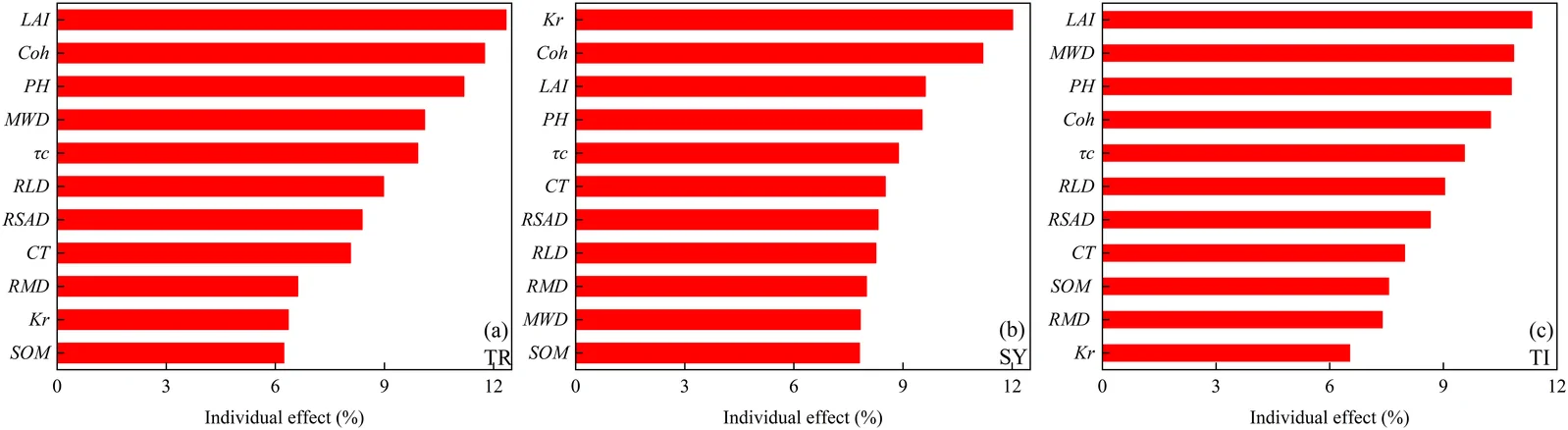
Individual effects of different soil properties, root traits, and vegetation features explaining the variations in the total runoff volume (*TR*) **(a)**, total sediment yield (*SY*) **(b)**, and total infiltration (*TI*) **(c)**.

Correlation analysis was conducted between the total runoff volume, sediment yield, and total infiltration and the near-soil surface traits (**Table 2**). No significant relationship was found between BD and total runoff volume, sediment yield, and total infiltration. The Coh, MWD, CT, *τ*_c_, RLD, RSAD, RMD, PH, and LAI were significantly negatively correlated with the total runoff volume and sediment yield and were significantly positively correlated with the total infiltration. Except for slope gradients of 17.63% and 26.79%, *K*_r_ was significantly positively correlated with the total runoff volume and sediment yield and was negatively correlated with the total infiltration. The correlation analysis results showed that the improvements in the near-soil surface traits significantly affected the soil erosion and reduced the soil erosion intensity. The interaction effects of soil properties, root system, and vegetation features accounted for the variations in the total runoff volume of 76.76%, sediment yield of 82.61%, and total infiltration of 85.71% (**Figure 8**). Among the near-soil surface traits, LAI had the highest individual contribution to the total runoff volume of 12.35% and the total infiltration of 11.34%, while *K*_r_ contributed to 12.02% of the variation in sediment yield (**Figure 9**).

**Table 2:** Correlation analysis between the near-soil surface characteristics and the indexes of the soil erosion process.

	Total runoff volume under slope gradients of 5.24%, 8.75%, 17.63%, 26.79%				Sediment yield under slope gradients of 5.24%, 8.75%, 17.63%, 26.79%				Total infiltration under slope gradients of 5.24%, 8.75%, 17.63%, 26.79%			
	TR3	TR5	TR10	TR15	SR3	SR5	SR10	SR15	TI3	TI5	TI10	TI15
BD	−0.266	−0.235	−0.309	−0.346	−0.217	−0.219	−0.226	−0.244	0.266	0.235	0.309	0.346
Coh	−0.984**	−0.979**	−0.980**	−0.966**	−0.954**	−0.962**	−0.966**	−0.967**	0.984**	0.979**	0.980**	0.966**
SOM	−0.845*	−0.841*	−0.803	−0.782	−0.884*	−0.886*	−0.889*	−0.877*	0.845*	0.841*	0.803	0.782
MWD	−0.968**	−0.958**	−0.982**	−0.949**	−0.840*	−0.853*	−0.861*	−0.872*	0.968**	0.958**	0.982**	0.949**
CT	−0.880*	−0.876*	−0.846*	−0.843*	−0.896*	−0.898*	−0.900*	−0.898*	0.880*	0.876*	0.846*	0.843*
*K*_r_	0.825*	0.815*	0.779	0.788	0.934**	0.930**	0.932**	0.917**	−0.825*	−0.815*	−0.779	−0.788
*τ*_c_	−0.961**	−0.950**	−0.951**	−0.970**	−0.922**	−0.925**	−0.930**	−0.943**	0.961**	0.950**	0.951**	0.970**
RLD	−0.960**	−0.938**	−0.957**	−0.984**	−0.927**	−0.929**	−0.936**	−0.950**	0.960**	0.938**	0.957**	0.984**
RSAD	−0.945**	−0.929**	−0.943**	−0.987**	−0.933**	−0.933**	−0.938**	−0.953**	0.945**	0.929**	0.943**	0.987**
RMD	−0.943**	−0.941**	−0.938**	−0.979**	−0.977**	−0.979**	−0.977**	−0.985**	0.943**	0.941**	0.938**	0.979**
PH	−0.991**	−0.977**	−0.987**	−0.988**	−0.938**	−0.944**	−0.950**	−0.960**	0.991**	0.977**	0.987**	0.988**
LAI	−0.984**	−0.979**	−0.987**	−0.997**	−0.926**	−0.933**	−0.936**	−0.950**	0.984**	0.979**	0.987**	0.997**

TR3, TR5, TR10, and TR15 represent the total runoff volume at slope gradients of 5.24%, 8.75%, 17.63%, and 26.79%, respectively. SR3, SR5, SR10, and SR15 represent the sediment yield at slope gradients of 5.5.24%, 8.75%, 17.63%, and 26.79%, respectively. TI3, TI5, TI10, and TI15 represent the total infiltration at slope gradients of 5.24%, 8.75%, 17.63%, and 26.79%, respectively.

*BD*, soil bulk density; *Coh*, soil cohesion; *SOM*, soil organic matter content; *MWD*, mean weight diameter; *CT*, crust thickness; *K*_r_, rill erodibility; *τ*_c_, critical shear stress; *RLD*, root length density; *RSAD*, root surface area density; *RMD*, root mass density; *PH*, plant height; *LAI*, leaf area index. *p < 0.05, ** p < 0.01.

## 4 Discussion

### 4.1 Seasonal variations in the near-soil surface traits

The near-soil surface traits presented obvious seasonal variations and increased over time, except for *K*_r_. This is consistent with the finding of Yao et al. (2025), who reported that plant growth significantly improved the near-surface characteristics and that *K*_r_ decreased over time with plant growth. Post-sowing, the soil structure was destroyed by tillage practice (Knapen et al., 2007b). The BD, Coh, and CT remained at a lower level. As millet grow, the soil was compressed by the roots, and fragmentation of the soil aggregates and rearrangement of the soil particles induced by raindrop impacts caused blockage of the soil pores, forming a layer that sealed the soil surface and leading to higher BD, CT, and Coh (Wang et al., 2025c; Yao et al., 2026). Furthermore, the formation of the root–soil complex also correspondingly led to an increase in Coh (Ma et al., 2022; Wang et al., 2025b). The adhesive substances, carbohydrates, phenols, and organic acids by root exudation facilitated the formation of water-stable aggregates and organic matter, thus leading to a progressive enhancement of the MWD and SOM (Daassi et al., 2024; Entezami et al., 2024; Saadati et al., 2023). The growth of crops promoted root system development and the increase of canopy coverage, thus resulting in an overall increase in the RLD, RSAD, RMD, and LAI (Wang et al., 2025c; Zeng et al., 2026). The shift from vegetative growth to reproductive growth from S4 to S5 resulted in the attenuation of root growth and the occurrence of dead roots, leading to the reduction in RLD and RSAD from S4 to S5 (Yao et al., 2022).

### 4.2 Soil erosion and its seasonal variation in sloping farmland

The measured soil erosion rate (RR and SR) for the millet cultivation plots showed temporal variations in all millet runoff plots and decreased over time during one entire growing season. The soil was disturbed by tillage operation at the early stage and a loose soil structure was formed, making the soil prone to erosion (Knapen et al., 2007b). Thus, the soil erosion rate was higher in S1. After sowing, the soil disturbance by tillage operation was rapidly eliminated by crop growth and dry–wet cycles (Knapen et al., 2007b; Yao et al., 2022). As reported by Chen et al. (2022b) and Yao et al. (2026), the soil erosion resistance significantly increased after the rainfall and the formation of soil crust. Consequently, the soil erosion rate decreased over time in the millet cultivation plots.

The measured soil erosion rate for the millet cultivation plots was noticeably lower than that for CK, which is likely the result of crop cultivation (Ma et al., 2021; Singh et al., 2025). This is consistent with the conclusion of Wang et al. (2025c) and Yao et al. (2022), who reported that crop cultivation and annual herbaceous plants significantly reduced the soil detachment intensity by changing the soil properties. In contrast to that for CK, the RRB and SRB for the millet cultivation plots were 4.16%–64.79% and 17.35%–93.18%, respectively. This result is consistent with Lin et al. (2019) and Yao et al. (2025), who reported that crop cultivation and the protective effects of herbaceous plants could effectively prolong the initial *T*_R_ and reduce soil erosion in the Loess Plateau.

### 4.3 Factors influencing soil erosion in sloping farmland

#### 4.3.1 Soil consolidation

**Figure 10:**
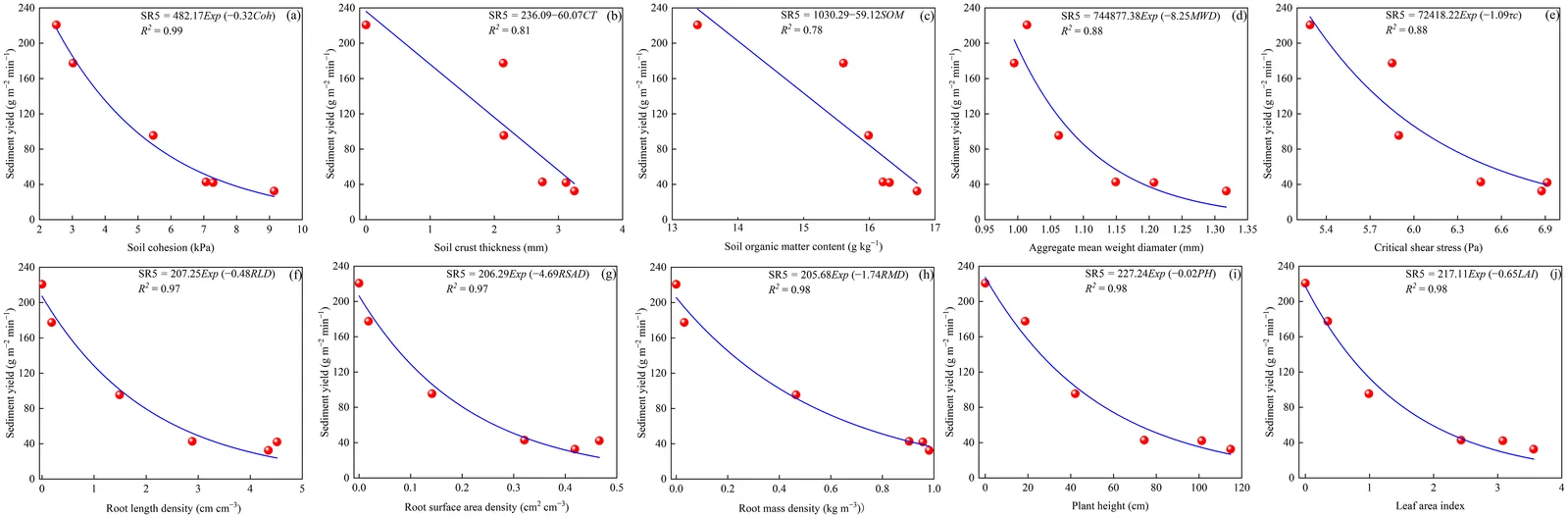
Relationship between sediment yield and near-soil surface traits: **(A)** soil cohesion (*Coh*), **(B)** crust thickness (*CT*), **(C)** soil organic matter content (SOM), **(D)** mean weight diameter (*MWD*), **(E)** critical shear stress (*τ*_c_), **(F)** root length density (*RLD*), **(G)** root surface area density (*RSAD*), **(H)** root mass density (*RMD*), **(I)** plant height (*PH*), and **(J)** leaf area index (*LAI*).

In the current study, the measured soil erosion rates decreased over time during one entire growing season. The sediment yield with a slope gradient of 8.75% was selected to explain the influence of crop growth on soil erosion in sloping farmland. The variation and the difference in soil erosion for the CK and millet cultivation plots were closely related to the variations in the soil properties induced by crop growth and the soil consolidation caused by precipitation (Chen et al., 2025; Knapen et al., 2007a; Yao et al., 2022). After cultivation, a dense soil layer was formed, leading to the changes in soil erosion resistance (De Baets et al., 2007; Knapen et al., 2007b; Wang et al., 2021; Yao et al., 2026). As observed on the soil properties, the increase in BD, Coh, and CT with crop growth reflected the soil consolidation process (Knapen et al., 2007b; Yao et al., 2022). The increase in BD reflected the increase in soil compaction, which was correspondingly accompanied by the enhancement of Coh, making the soil mass more resistant to runoff scouring (Knapen et al., 2007b; Parhizkar et al., 2025; Wang et al., 2025c). In the current study, the enhanced Coh induced an exponential decrease in sediment yield (**Figure 10A**). This is supported by Wang et al. (2025b), who reported that the increase in cohesion enhanced soil erosion resistance and reduced rill erosion. The splashed fine soil particles by raindrops filled the soil porosity, advancing the formation of soil crusts and making the soil more compact and difficult to detach (Chen et al., 2022a, 2022b; Lu et al., 2017; Zeng et al., 2026). Consequently, the sediment yield decreased with the increase in CT (**Figure 10B**).

#### 4.3.2 Soil stability

SOM and MWD were used to indicate soil stability and aggregate stability, respectively (Barbosa et al., 2025; Wang et al., 2025b; Zhang et al., 2025b). Substances such as polysaccharides, amino acids, and organic acids are secreted by the root system, promoting the formation of organic–inorganic complexes and providing carbon sources for rhizosphere microorganisms (Daassi et al., 2024; Saadati et al., 2023). Soil microorganisms enhance the stability of the soil water-stable aggregates by hyphal entanglement and the secretion of extracellular adhesive substances (Entezami et al., 2024; Saadati et al., 2023). In this study, the MWD and SOM increased with crop growth throughout the entire growing season. Root exudates containing sugars, organic acids, and phenols are conducive to the formation of water-stable aggregates and the accumulation of SOM (Shuai et al., 2025; Yao et al., 2023; Zhang et al., 2025a; Zi et al., 2023). Thus, the sediment yield was negatively correlated with the SOM and MWD (**Figures 10C, D**). This result is consistent with Vahidi et al. (2025), who reported that organic amendments enhanced the soil stability and increased the soil erosion resistance, consequently reducing soil erosion. The *τ*_c_ characterizes the critical condition for soil detachment by runoff. In this study, the sediment yield decreased with the *τ*_c_ as an exponential function (**Figure 10E**). With crop growth, the formation of root–soil complexes increases the *τ*_c_ and reduces the potential for soil erosion (Shuai et al., 2025; Wang et al., 2025b; Zhu et al., 2026).

#### 4.3.3 Root system

Crop roots generally influence the soil erosion process through bonding and binding effects (De Baets et al., 2007; Wang and Zhang, 2017; Zhu et al., 2026). During the seedling stage of crops, the root density is low and the ability to fix the soil is weak. Coupled with the raindrop impact and runoff scouring in the rainy season, the risk of soil erosion is higher. In the middle growth stage of crops, the soil–root stabilizing network is formed by a dense shallow root system, slowing down runoff and increasing the soil IR, thus minimizing soil erosion to the greatest extent. During the later stage of crop growth, the change in root density is relatively small, resulting in a minor change in the soil erosion rate. The regulatory and mitigating effects of plant roots on soil erosion are generally determined by the length, surface area, and mass of the roots (Shuai et al., 2025; Wang et al., 2021, 2026; Zhang et al., 2025a). In general, root length characterizes the length of the root system that is interspersed in the soil (Wang et al., 2025b; Yao et al., 2022). A longer root distribution in the soil enhances soil fixation and protection, making the soil more resistant to runoff erosion (Wang et al., 2025a; Zhu et al., 2026). According to the results of this study, RLD has an exponential function relationship with the sediment yield (**Figure 10F**). The RSAD indicates the contact area between the root and the soil (De Baets et al., 2007; Yao et al., 2022). A larger root surface area is conducive to promoting the release of root exudates into the soil and is beneficial for enhancing the adhesive effect of the roots (Wang and Zhang, 2017). According to our research results, the soil erosion rate showed an exponential function decrease with the increase of RSAD (**Figure 10G**). The root mass reflects the number of roots in the soil (Barbosa et al., 2025; Zhou et al., 2025). The increase in the RMD is conducive to the stabilization and formation of the root–soil complex. An exponential function relationship was found between the sediment yield and RMD (**Figure 10H**). This is supported by Yao et al. (2023), who reported that the plant roots showed a superior capacity to reduce runoff and sediment yield. The results of this study demonstrated a relatively small number of roots and a high soil erosion rate in the early stage of crop growth. The soil erosion rate decreased with root system development, indicating that root system development was conducive to reducing soil erosion.

#### 4.3.4 Vegetation features

The regulation efficiency of the vegetation aboveground components on soil erosion processes is realized by increasing the soil surface roughness and exerting shielding effects (Di Prima et al., 2026; Fu et al., 2023). The impact of the aboveground components on soil erosion could be determined by the vegetation coverage, PH, and LAI (Qiu et al., 2025; Yao et al., 2025). Plants could promote infiltration and reduce soil erosion by increasing stem flow (Di Prima et al., 2026; Levia and Frost, 2003). The results showed that the sediment yield decreased exponentially with the increase in PH (**Figure 10I**). Aboveground vegetation protects the soil by shielding rainfall and reducing the direct impact of raindrops (Peng et al., 2026; Song et al., 2026; Tuo et al., 2025). In general, a higher LAI indicates a higher vegetation coverage, which reduces the potential for soil erosion occurring (Feng and Jin, 2024; Gu et al., 2025; Wang et al., 2024). In the current study, the sediment yield decreased with the increase in LAI as an exponential function (**Figure 10J**). Our results are in agreement with Yao et al. (2023), who reported that the sediment yield decreased exponentially with the aboveground biomass.

### 4.4 Implication of the current study

In this study, rainfall simulation was conducted on both millet cultivation and CK plots. Seasonal variations in soil erosion processes and their response to near-soil surface traits were investigated. The results demonstrated that soil loss in sloping farmland did not remain at a high level throughout the growing season. The soil erosion rate remained higher after sowing due to soil disturbance. As crops grew, the soil properties, root systems, and vegetation characteristics changed, increasing soil erosion resistance, correspondingly reducing the soil erosion rate. The reduction in the soil erosion rate is closely related to the combined effects of the near-soil surface features rather than solely on the soil properties or vegetation characteristics. The results from this study provide strategies for innovating soil and water conservation measures to reduce soil erosion on sloping farmland in the Loess Plateau. In future research, seasonal variations in the nitrogen, phosphorus, and potassium carried by sediment and runoff from sloping farmland should be investigated. The adoption of reasonable measures is beneficial to preventing land degradation, increasing grain production, and ensuring soil sustainability.

## 5 Conclusion

In the current study, rainfall simulation was carried out in CK and millet cultivation plots at different crop growth stages. During one growing season, the BD, SOM, and *τ*_c_, with variation coefficients of 0.03–0.08, demonstrated weak variability; the MWD, Coh, RLD, RSAD, RMD, PH, and LAI, with variation coefficients of 0.11–69, indicated moderate variability; and *K*_r_, with a variation coefficient of 1.26, showed strong variability. The time to runoff initiation for CK ranged from 1.40 to 5.25 min, while that for millet cultivation ranged from 3.40 to 14.30 min, which advanced with increasing slope gradient and was delayed with crop growth. The mean RR, SR, and IR in the millet cultivation plots ranged 0.32–0.92 L m^−2^ min^−1^, 0.64–6.16 g m^−2^ min^−1^, and 0.43–1.09 mm m^−2^ min^−1^, respectively. The RRB and SRB, which were 4.16%–64.79% and 17.35%–93.18%, respectively, increased with crop growth and decreased with slope gradient. The interaction effects of the soil properties, the root system, and the vegetation features accounted for the variations in the total runoff volume of 76.76%, sediment yield of 82.61%, and total infiltration of 85.71%. Among the near-soil surface traits, LAI made the highest individual contribution to the variations in the total runoff volume of 12.35% and the total infiltration of 11.34%, while *K*_r_ contributed to the variation in sediment of 12.02%. Overall, the Coh, SOM, MWD, CT, *τ*_c_, RLD, RSAD, RMD, PH, and LAI were significantly negatively correlated with the total runoff volume and sediment yield and significantly positively correlated with the total infiltration. The results demonstrated the influence of changes in the near-soil surface traits induced by crop cultivation on the seasonal variations in soil erosion, providing scientific references for the prevention of soil erosion in sloping farmland.

## Data Availability

The original contributions presented in the study are included in the article/supplementary material. Further inquiries can be directed to the corresponding author.

## References

[B1] Bahddou S. Otten W. Whalley W. R. Shin H.-C. El Gharous M. Rickson R. J. (2023). Changes in soil surface properties under simulated rainfall and the effect of surface roughness on runoff, infiltration and soil loss. Geoderma 431, 116341. doi: 10.1016/j.geoderma.2023.116341 38826717

[B2] Barbosa L. A. P. Leue M. Wehrhan M. Sommer M. (2025). Impact of wheat cultivar development on biomass and subsoil carbon input: a case study along an erosion-deposition gradient. Biogeosciences 22, 5651–5664. doi: 10.5194/bg-22-5651-2025

[B3] Bautista S. Mayor Á.G. Bourakhouadar J. Bellot J. (2007). Plant spatial pattern predicts hillslope runoff and erosion in a semiarid Mediterranean landscape. Ecosystems 10, 987–998. doi: 10.1007/s10021-007-9074-3 30311153

[B4] Burylo M. Rey F. Mathys N. Dutoit T. (2012). Plant root traits affecting the resistance of soils to concentrated flow erosion. Earth Surf. Processes Landforms 37, 1463–1470. doi: 10.1002/esp.3248 41531421

[B5] Chen L. Wang J. Wang H. Xu F. F. Song P. S. Yang C. . (2022a). Variation in soil detachment capacity of structural and sedimentary crusts induced by simulated rainfall formed on ridge and furrow. Catena 211, 105971. doi: 10.1016/j.catena.2021.105971 38826717

[B6] Chen L. Xu F. Li J. Yang C. Wang J. (2022b). Aggregate stability in rainfall-induced soil physical crusts on the Loess Plateau, Northwest China. Soil Sci. Soc Am. J. 86, 528–539. doi: 10.1002/saj2.20389 41531421

[B7] Chen S. Zhang G. Wang C. (2025). Temporal variation in soil erodibility indicators of sloping croplands with different straw-incorporation rates. Soil Tillage Res. 246, 106340. doi: 10.1016/j.still.2024.106340 38826717

[B8] Chen Y. Qi Y. Wei Y. Ning W. H. He B. H. (2024). Root traits and soil detachment in response to variable slope gradients in a representative purple-soil sloping grassland. Catena 239, 107936. doi: 10.1016/j.catena.2024.107936 38826717

[B9] Daassi R. Khasa D. P. Stevanovic T. (2024). Effect of ramial chipped wood and poultry manure amendments on soil chemical properties and fungal communities in Benin. Soil Tillage Res. 237, 105974. doi: 10.1016/j.still.2023.105974 38826717

[B10] De Baets S. Poesen J. Knapen A. Galindo P. (2007). Impact of root architecture on the erosion‐reducing potential of roots during concentrated flow. Earth Surf. Processes Landforms 32, 1323–1345. doi: 10.1002/esp.1470 41531421

[B11] Di Prima S. Fernandes G. Burguet M. Salazar M. P. Marras E. Murgia I. . (2026). Trees control hillslope subsurface flow: Insights from stemflow and throughfall experiments, geophysical surveys, and numerical modeling. J. Hydrol. 665, 134723. doi: 10.1016/j.jhydrol.2025.134723 38826717

[B12] Entezami E. Mosaddeghi M. R. Shirvani M. Khalili B. Bazarganipour M. (2024). Interactive effects of Ag nanoparticles/nitrate and plant root systems on quality indicators and aggregate stability of two texturally-different soils. Soil Tillage Res. 244, 106257. doi: 10.1016/j.still.2024.106257 38826717

[B13] Feng H. J. Jin J. M. (2024). Responses of vegetation to hydroclimatic variables on the Loess Plateau after large scale vegetation restoration. Hydrol. Processes 38, e15283. doi: 10.1002/hyp.15283 41531421

[B14] Fu Y. Wang D. Sun W. Guo M. (2023). Impacts of grass planting density and components on overland flow hydraulics and soil loss. Land Degrad. Dev. 34, 234–249. doi: 10.1002/ldr.4456 41531421

[B15] Futerman S. I. Cohen Y. Laor Y. Argaman E. Aharon S. Eshel G. (2025). Assessing field-scale rill erosion mitigation by cover crops in arable land using drone image analysis. Soil Tillage Res. 246, 106341. doi: 10.1016/j.still.2024.106341 38826717

[B16] Geng R. Zhang G. Li Z.-W. Wang H. (2015). Spatial variation in soil resistance to flowing water erosion along a regional transect in the Loess Plateau. Earth Surf. Processes Landforms 40, 2049–2058. doi: 10.1002/esp.3779 41531421

[B17] Gu H. Wang Y. Liu S. (2025). Interspecific interactions enhance soil resistance to erosion: Synergistic effects of mixed grass species under simulated rainfall. J. Hydrol. 660, 133452. doi: 10.1016/j.jhydrol.2025.133452 38826717

[B18] Gyssels G. Poesen J. Bochet E. Li Y. (2005). Impact of plant roots on the resistance of soils to erosion by water: a review. Prog. Phys. Geogr. 29, 189–217. doi: 10.1191/0309133305pp443ra 19957548

[B19] Jiang L. S. Tan X. H. Xia Y. Y. Gan F. L. Xu X. Y. Pu J. B. . (2025). The responses of soil erosion resistance to the farmland abandonment of bedding/inverse slopes in karst trough valley. Soil Tillage Res. 252, 106577. doi: 10.1016/j.still.2025.106577 38826717

[B20] Jimenez Aguilar A. Huber-Sannwald E. Belnap J. Smart D. R. Arredondo Moreno J. T. (2009). Biological soil crusts exhibit a dynamic response to seasonal rain and release from grazing with implications for soil stability. J. Arid. Environ. 73, 1158–1169. doi: 10.1016/j.jaridenv.2009.05.009 38826717

[B21] Knapen A. Poesen J. Baets S. D. (2007a). Seasonal variations in soil erosion resistance during concentrated flow for a loess-derived soil under two contrasting tillage practices. Soil Tillage Res. 94, 425–440. doi: 10.1016/j.still.2006.09.005 38826717

[B22] Knapen A. Poesen J. Govers G. Gyssels G. Nachtergaele J. (2007b). Resistance of soils to concentrated flow erosion: A review. Earth Sci. Rev. 80, 75–109. doi: 10.1016/j.earscirev.2006.08.001 38826717

[B23] Levia D. F. Frost E. E. (2003). A review and evaluation of stemflow literature in the hydrologic and biogeochemical cycles of forested and agricultural ecosystems. J. Hydrol. 274, 1–29. doi: 10.1016/S0022-1694(02)00399-2

[B24] Li Q. Liu G. B. Zhang Z. Tuo D. F. Bai R. R. Qiao F. F. (2017). Relative contribution of root physical enlacing and biochemistrical exudates to soil erosion resistance in the Loess soil. Catena 153, 61–65. doi: 10.1016/j.catena.2017.01.037 38826717

[B25] Li Z. Zhang G. Geng R. Wang H. (2015). Rill erodibility as influenced by soil and land use in a small watershed of the Loess Plateau, China. Biosyst. Eng. 129, 248–257. doi: 10.1016/j.biosystemseng.2014.11.002 38826717

[B26] Lin Q. T. Xu Q. Wu F. Q. Li T. T. (2019). Effects of wheat in regulating runoff and sediment on different slope gradients and under different rainfall intensities. Catena 183, 104196. doi: 10.1016/j.catena.2019.104196 38826717

[B27] Loades K. W. Bengough A. G. Bransby M. F. Hallett P. D. (2010). Planting density influence on fibrous root reinforcement of soils. Ecol. Eng. 36, 276–284. doi: 10.1016/j.ecoleng.2009.02.005 38826717

[B28] Lu P. Xie X. L. Wang L. H. Wu F. Q. (2017). Effects of different spatial distributions of physical soil crusts on runoff and erosion on the Loess Plateau in China. Earth Surf. Processes Landforms 42, 2082–2089. doi: 10.1002/esp.4175 41531421

[B29] Ludwig J. A. Eager R. W. Liedloff A. C. Bastin G. N. Chewings V. H. (2006). A new landscape leakiness index based on remotely sensed ground-cover data. Ecol. Indic. 6, 327–336. doi: 10.1016/j.ecolind.2005.03.010 38826717

[B30] Ma B. Li C. D. Li Z. B. Wu F. Q. (2016). Effects of crops on runoff and soil loss on sloping farmland under simulated rainfall. CLEAN - Soil Air Water 44, 849–857. doi: 10.1002/clen.201400241 41531421

[B31] Ma B. Liu Y. X. Liu X. J. Ma F. Wu F. Q. Li Z. B. (2015). Soil splash detachment and its spatial distribution under corn and soybean cover. Catena 127, 142–151. doi: 10.1016/j.catena.2014.11.009 38826717

[B32] Ma J. Li Z. Ma B. Wang C. Sun B. Shang Y. (2022). Response mechanism of the soil detachment capacity of root-soil composites across different land uses. Soil Tillage Res. 224, 105501. doi: 10.1016/j.still.2022.105501 38826717

[B33] Ma R. Zheng Z. C. Li T. X. He S. Q. Zhang X. Z. Wang Y. D. . (2021). Temporal variation of soil erosion resistance on sloping farmland during the growth stages of maize (Zea mays L.). Hydrol. Processes 35, e14353. doi: 10.1002/hyp.14353 41531421

[B34] Nandintsetseg B. Shinoda M. (2011). Seasonal change of soil moisture in Mongolia: its climatology and modelling. Int. J. Climatol. 31, 1143–1154. doi: 10.1002/joc.2134 41531421

[B35] Nardi S. Concheri G. Pizzeghello D. Sturaro A. Rella R. Parvoli G. (2000). Soil organic matter mobilization by root exudates. Chemosphere 41, 653–658. doi: 10.1016/S0045-6535(99)00488-9 10834364

[B36] Parhizkar M. Lucas-Borja M. E. Filianoti P. G. F. Tziolas N. Zupanc V. Zema D. A. (2025). Effects of treatments with biochar from crop residues on soil detachment capacity in rills after deforestation. Soil Use Manage. 41, e70070. doi: 10.1111/sum.70070 40046247

[B37] Peng D. Q. Du C. H. Xu R. L. Liu J. L. Zhang X. Y. Guo M. M. . (2026). Sustainable soil management: Rhizosphere microbial contributions to erosion control in herbaceous vegetation systems. Soil Tillage Res. 256, 106844. doi: 10.1016/j.still.2025.106844 38826717

[B38] Puigdefábregas J. (2005). The role of vegetation patterns in structuring runoff and sediment fluxes in drylands. Earth Surf. Processes Landforms 30, 133–147. doi: 10.1002/esp.1181 41531421

[B39] Qiu M. Pei X. J. Zhang R. J. Zhang X. C. Xi H. C. Zhao X. Y. . (2025). Vegetation restoration affects soil erosion processes by altering the soil net force. Catena 251, 108823. doi: 10.1016/j.catena.2025.108823 38826717

[B40] Quinton J. N. Govers G. Van. Oost K. Bardgett R. D. (2010). The impact of agricultural soil erosion on biogeochemical cycling. Nat. Geosci. 3, 311–314. doi: 10.1038/ngeo838 37880705

[B41] Saadati N. Mosaddeghi M. R. Sabzalian M. R. Jafari M. (2023). Soil mechanical reinforcement by the fibrous roots of selected rangeland plants using a large soil-root shear apparatus. Soil Tillage Res. 234, 105852. doi: 10.1016/j.still.2023.105852 38826717

[B42] Shuai F. Wu W. Meng Y. Zhou Y. Y. Chen Y. Y. Zhang Y. . (2025). Effect of shrub root diameter classes on shear strength of soil in Benggang collapsing walls. Earth Surf. Processes Landforms 50, e70128. doi: 10.1002/esp.70128 41531421

[B43] Singh R. J. Kumar G. Sharma N. K. Deshwal J. S. Mishra M. Roy P. . (2025). Conservation tillage-based Arundo donax agro-geotextiles enhance productivity and profitability of sloping croplands in the Indian Himalayas by reducing soil erosion and improving soil organic carbon. J. Environ. Manage. 377, 124728. doi: 10.1016/j.jenvman.2025.124728 40024156

[B44] Song Y. Yao Y. F. Kong W. B. Guo L. C. Bao K. Q. Qiu L. P. . (2026). Effects of vegetation loss and soil erosion intensity on soil carbon dynamics across landscape position: Evidence from China's Loess Plateau. Agric. Ecosyst. Environ. 396, 109992. doi: 10.1016/j.agee.2025.109992 38826717

[B45] Tuo M. Qiao H. L. Xu G. C. Wang B. Wan S. Wang X. N. . (2025). Effects of vegetation types on hillslope runoff and soil erosion on the Loess Plateau. Catena 260, 109487. doi: 10.1016/j.catena.2025.109487 38826717

[B46] Vahidi M. Bayat H. Ebrahimi M. (2025). Evaluating the impact of organic amendments on soil erosion dynamics: a comprehensive examination of application methods and timing. Can. J. Soil Sci. 105. doi: 10.1139/cjss-2025-0032 34819996

[B47] Van Dijk A. I. J. M. Bruijnzeel L. A. (2001). Modelling rainfall interception by vegetation of variable density using an adapted analytical model. Part 2. Model validation for a tropical upland mixed cropping system. J. Hydrol. 247, 239–262. doi: 10.1016/S0022-1694(01)00392-4

[B48] Vannoppen W. Vanmaercke M. De Baets S. Poesen J. (2015). A review of the mechanical effects of plant roots on concentrated flow erosion rates. Earth Sci. Rev. 150, 666–678. doi: 10.1016/j.earscirev.2015.08.011 38826717

[B49] Wang X. Chen X. Q. Chen H. Y. Zhao W. Y. (2024). Influence mechanism of herbaceous plants on debris flow bank erosion. Catena 245, 108308. doi: 10.1016/j.catena.2024.108308 38826717

[B50] Wang L. H. Dalabay N. Lu P. Wu F. Q. (2017). Effects of tillage practices and slope on runoff and erosion of soil from the Loess Plateau, China, subjected to simulated rainfall. Soil Tillage Res. 166, 147–156. doi: 10.1016/j.still.2016.09.007 38826717

[B51] Wang B. Li P. P. Huang C. H. Liu G. B. Yang Y. F. (2021). Effects of root morphological traits on soil detachment for ten herbaceous species in the Loess Plateau. Sci. Total Environ. 754, 142304. doi: 10.1016/j.scitotenv.2020.142304 33254931

[B52] Wang J. F. Liu G. B. Yang Y. F. Wang B. (2026). Construction of a comprehensive root parameter to reflect the effect of plant root systems on soil erosion. Land Degrad. Dev. 36, 4359–4371. doi: 10.1002/ldr.70456 41531421

[B53] Wang C. Ma J. Wang Y. Li Z. Ma B. (2022). The influence of wheat straw mulching and straw length on infiltration, runoff and soil loss. Hydrol. Processes 36, e14561. doi: 10.1002/hyp.14561 41531421

[B54] Wang J. F. Yang Y. F. Wang B. Liu G. B. (2025a). Distinguishing the effects of vegetation characteristics on soil erosion process on the loess plateau of China. Earth Surf. Processes Landforms 50, e70011. doi: 10.1002/esp.70011 41531421

[B55] Wang J. F. Yang Y. F. Wang B. Liu G. B. (2025b). Effects of vegetation restoration on rill erosion on the Chinese Loess Plateau. Land Degrad. Dev. 36, 4359–4371. doi: 10.1002/ldr.5639 41531421

[B56] Wang J. F. Yang Y. F. Wang B. Liu G. B. (2025c). Seasonal variations in soil erosion resistance under tap and fibrous root systems grasslands on the Chinese Loess Plateau. Geoderma 458, 117350. doi: 10.1016/j.geoderma.2025.117350 38826717

[B57] Wang B. Zhang G. H. (2017). Quantifying the binding and bonding effects of plant roots on soil detachment by overland flow in 10 typical grasslands on the Loess Plateau. Soil Sci. Soc Am. J. 81, 1567–1576. doi: 10.2136/sssaj2017.07.0249

[B58] Wang C. Zhang X. Wang Y. Ma B. (2023). Effects of straw mixed mulch length and coverage on infiltration, soil and water loss of Loess Plateau slopes. Land Degrad. Dev. 34, 2931–2944. doi: 10.1002/ldr.4657 41531421

[B59] Yao C. Chen K. Zhang Q. (2023). The contribution rate of stem-leaf and root of alfalfa (Medicago sativa L.) to sediment and runoff reduction. Land Degrad. Dev. 34, 34. doi: 10.1002/ldr.4731 41531421

[B60] Yao C. Ye S. Z. Chen S. Y. Zhang M. J. Zhu M. Wu F. Q. (2025). The effect of soybean growth on the seasonal variation of hillslopes erosion in gently sloping farmland on the Loess Plateau. Land Degrad. Dev. 36, 3975–3988. doi: 10.1002/ldr.5611 41531421

[B61] Yao C. Zhang Q. W. Lu C. Li H. K. Wang H. Wu F. Q. (2022). Variations in soil detachment by rill flow during crop growth stages in sloping farmlands on the Loess Plateau. Catena 216, 106375. doi: 10.1016/j.catena.2022.106375 38826717

[B62] Yao C. Zhang Q. Zhuan Y. Fu S. Lin H. Wang H. . (2026). Rainfall-induced soil physical crust formation reduces soil detachment capacity by enhancing soil erosion resistance. Land Degrad. Dev. n/a. doi: 10.1002/ldr.70433 41531421

[B63] Yoder R. E. (1936). A direct method of aggregate analysis of soils and a study of the physical nature of erosion losses. J. Am. Soc. Agron. 28, 337–351. doi: 10.2134/agronj1936.00021962002800050001x

[B64] Zeng J. H. Guo Z. L. Li D. Y. Wei Y. J. Liu F. J. Cai C. F. . (2026). Regulation pathways of physical crusts on seasonal dynamics of soil detachment capacity under different ridging patterns. Soil Tillage Res. 255, 106823. doi: 10.1016/j.still.2025.106823 38826717

[B65] Zhang Z. Li Q. Liu G. B. Tuo D. F. J. (2017). Soil resistance to concentrated flow and sediment yields following cropland abandonment on the Loess Plateau, China. J. Soils Sediments 17, 1662–1671. doi: 10.1007/s11368-017-1650-3 30311153

[B66] Zhang Y. Pang Z. L. Chen J. Chen X. W. Wang E. H. (2025a). Root-mediated regulation of soil infiltration dynamics in eroded Mollisols. Geoderma 462, 117508. doi: 10.1016/j.geoderma.2025.117508 38826717

[B67] Zhang Y. T. Zhang G. H. Zhang N. Xing S. K. Zhang Y. (2025b). Response of soil erosion resistance to incorporated straw affected by different microbial inoculants in the black soil region of Northeast China. Agric. Ecosyst. Environ. 392, 109675. doi: 10.1016/j.agee.2025.109675 38826717

[B68] Zhao L. Meng P. Zhang J. Zhang J. Li J. Wang X. (2023). The contribution of human activities to runoff and sediment changes in the Mang River basin of the Loess Plateau, China. Land Degrad. Dev. 34, 28–41. doi: 10.1002/ldr.4441 41531421

[B69] Zhou Z. C. Zheng Q. W. Chen M. Y. Wang N. Liu J. Zhu B. B. (2025). Response of soil detachment and erodibility to perennial fibrous-rooted vegetation coverage (Stipa bungeana) on the Loess Plateau. Catena 255, 109043. doi: 10.1016/j.catena.2025.109043 38826717

[B70] Zhu R. P. Zhang G. H. Xing S. K. (2026). Soil-root shear strength of gullies covered by different vegetation types on the Loess Plateau of China. Land Degrad. Dev. doi: 10.1002/ldr.70435 41531421

[B71] Zi R. Zhao L. Fang Q. Qian X. Fang F. Fan C. (2023). Path analysis of the effects of hydraulic conditions, soil properties and plant roots on the soil detachment capacity of karst hillslopes. Catena 228, 107177. doi: 10.1016/j.catena.2023.107177 38826717

